# Research on Spatio-Temporal Characteristics of Tourists’ Landscape Perception and Emotional Experience by Using Photo Data Mining

**DOI:** 10.3390/ijerph20053843

**Published:** 2023-02-21

**Authors:** Junxia Yan, Jiaheng Yue, Jianfeng Zhang, Peng Qin

**Affiliations:** 1 College of Geographical Sciences, Hebei Normal University, Shijiazhuang 050024, China; 2 Department of Geography, Handan College, Handan 056005, China; 3 Institute of Geological Survey, China University of Geosciences, Wuhan 430074, China; 4 College of Resources and Environment, Qingdao Agricultural University, Qingdao 266109, China

**Keywords:** visual semantics, DeepSentiBank, location photos, landscape perception, quantization methods

## Abstract

Mountainous scenic spots are important tourism resources, and the study of tourists’ landscape perception and emotional preference when visiting them is beneficial to the management of scenic spots in order to improve the service quality and promote the protection, development, and utilization of scenic resources. In this paper, we use the location photo data of tourists at Huangshan Mountain to extract the visual semantic information of location photos, calculate the photo sentiment value, and mine the landscape perception and sentiment preference features of tourists using DeepSentiBank image recognition model and photo visual semantic quantification method. The results show the following: (1) Huangshan tourists mainly focus on nine types of photos, with the most attention paid to the category of mountain rock landscapes and the least attention paid to the category of animal landscapes. (2) In terms of spatial distribution, the landscape types of tourist photos show the spatial characteristics of “concentrated into a belt”, “significant nucleus”, and “fragmented distribution”. The spatial variation of the emotional value of tourists’ photos is significant, and the high values are mainly distributed at the entrances and exits, interchanges, and famous attractions. (3) On a temporal scale, the type of perception of the Huangshan location photograph landscape shows a significant imbalance. The emotional values of tourists’ photos vary significantly, with a “slowly sloping straight line” type of emotional change on the seasonal scale, a “W” type of emotional change on the monthly scale, an “N” type of emotional change on the weekly scale, and an “M” type of emotional change on the hourly scale. This study attempts to explore the landscape perceptions and emotional preferences of tourists in mountainous scenic areas with new data and methods, aiming to promote the sustainable and high-quality development of mountainous scenic areas.

## 1. Introduction

At present, most studies are based on spatial and attributed information from text. Compared with text data, location photo data has rich visual semantic information, which can more intuitively reflect the interests of tourists, and it is important to explore the visual information of photos for the study of tourists’ landscape perception and emotional preferences. This can have a positive effect on the ability of tourism management departments to improve scenic services according to tourists’ landscape perception and emotional preferences and accelerate the recovery and development of tourist attractions after the COVID-19 pandemic [1,2].

Photographs are a record of tourists’ interest in the landscape, and taking them is an important activity for tourists when traveling [3]. With the development of social network platforms, a huge amount of UGC (user-generated content) data has been generated. Tourists post photos, travelogues, and Weibo on Flickr, “Sina Weibo”, “2BULU”, “Six Feet”, and other domestic and international web interaction platforms, forming huge amounts of UGC data, mainly in the form of text and images, which provide a rich database for tourism-related research and material for tourism marketing organizations to focus on different types of tourists and design marketing campaigns. The photo data uploaded by tourists come from the photo-taking activities during their trips, and uploading through mobile devices ensures that photos are accompanied by EXIF information, such as the location and time of shooting [4]. Photos can express tourists’ instinctive perceptions of destinations and reflect their potential attitudes and intentions. Using tourists’ photo data locations to carry out relevant research is of practical significance to exploring tourists’ travel behavior, landscape perception, and emotional preferences.

At present, most studies on landscape perception are based on remote sensing data analyses. Adison et al. [5] used remote sensing data to evaluate tourists’ landscape perceptions and preferences in the southern Chilean landscape. Li et al. [6] conducted a study on landscape perception calculation for the Wu’an National Geopark, using remote sensing data. Relatively speaking, the UGC data-based landscape perception studies of tourists are still in the minority. The location photos are a visual and emotional expression of the tourist’s view and feelings about a touristic location and contain rich visual semantic information, as well as having different spatial and temporal scale characteristics, which can intuitively reflect what the tourist “sees” and “feels” and also reflect the tourist’s landscape perception and emotional preference of “where”. Pan et al. [7] used location photo data to study motivation, image dimensions, and the affective qualities of tourist destinations, and Stepchenkova et al. [8] used Flickr photos to construct a landscape perception map representing Peru. Mining and analyzing the semantic information of location photos and combining the visual content of photos with spatial and temporal scales is an effective way to analyze tourists’ spatial and temporal behaviors and landscape attention types and explore tourists’ landscape perceptions and emotional preferences [9]. In the past, due to the limitations of visual semantic mining technology, most of the research on the visual content of photos was based on manual recognition and classification coding methods, which were inefficient in processing data volume, and the research results were also extremely subjective [10]. Along with the rapid development of computer deep learning and big data mining technology, computer image processing technology is becoming more and more mature, and artificial intelligence is widely used in various fields of image recognition [11]. The use of artificial intelligence big data processing methods to identify and parse the contents of tourists’ location photos breaks through the limitations of manual methods and provides technical support for mining the complex visual semantic information of these images [8]. When applying location photo visual semantic mining technology to the study of tourists’ landscape perceptions and emotion analysis, on the one hand, the visual semantics mined from tourists’ location photos are used to classify landscape perception types, and on the other hand, the emotional preferences of tourists’ landscape perception at different spatial and temporal scales are summarized. In this paper, we use deep learning technology to analyze the visual semantics of tourists’ photos from Huangshan Mountain and use the location and time information contained in the photos to summarize the types of landscape perceptions and the high and low sentiment values of tourists’ photos through various time scales, namely seasons, months, days of the week, and hours, and various spatial scales, namely scenic spots and scenic sections, and analyze and condense the landscape perception characteristics and the sentiment preferences of tourists.

## 2. Literature Review

In early studies, Cherem, Haywood, et al. used the tourist-employed photograph (VEP) method to collect photographic data representing tourists’ subjective experiences in order to analyze travelers’ experiences of natural environments and cities from a touristic point of view [12,13]. Since the beginning of the 21st century, Huang [14] and others used the VEP method to summarize the factors influencing tourists’ scenic experiences and the components of place attachment in tourist destinations, respectively, while Zong [15] explored the imagery of Fuzhou by collecting photos through the VEP method and combining it with photo content coding. Such methods provide ideas for the qualitative research of tourists’ photos; however, there are problems such as limited amounts of data, a high cost of obtaining the data, and a high workload.

Mining the visual content of tourism photos is predominantly based on the results of the manual recognition of images in early related studies, such as Hunter, who used a content analysis method to study the web image of Seoul, Korea [16], and Zheng, who constructed the visual representation of the tourism location image using the NViov10 qualitative coding method [17]. Yang [18] and Wu [19] used geo-tagged photos to study the spatial characteristics of tourism flows; Shen [20] and others conducted tourist behavior research based on geo-photo metadata; Kuo [21] and others used the geographical information of location photos combined with spatial analysis methods to explore the discovery and extraction of POI (point of interest) and AOI (region of interest) method; and Mou [22] also explored the spatial and temporal variation of urban inbound tourism flows based on this method. Deng et al. [23] combined the textual information in photo metadata, which is usually viewed as tourist perception content, with imagery studies and utilized the label comment information in picture metadata as a mapping of the cognitive and affective images of pictures, so as to indirectly analyze the image of tourist locations. The development of computer technology enabled the possibility of the machine recognition of a huge number of pictures, and tourism-related studies based on the deep learning recognition of photos began to emerge. Kang et al. [24] analyzed the cityscape image of Seoul using the Inception-v3 model and Flickr data. Zhang et al. [25] performed scene recognition on photos of tourists in Beijing to compare the behavioral and cognitive differences of tourists without using countries. The study by Deng et al. [26] is based on the deep learning analysis of pictures to explore different tourist destination image perceptions. Cao et al. [27] conducted a comparative study on the imagery of 24 major cities in China based on machine-tagged text from Flickr photo metadata. Bubalo et al. [28] collected geographical information about landscape perception and preferences by summarizing different crowdsourcing models.

Landscape perception refers to the process of interaction between people and the landscape, where perceptions are formed in the course of experiential activities, and the results of perceptions, in turn, influence people and the landscape itself. The content of landscape perception includes landscape perception types, landscape preferences and attitudes, and landscape value perception. Using the visual content of photographs to analyze the landscape perceptions and emotional preferences of tourists in tourism destinations has become a good research direction. Dunkel [29] uses Flickr photo data to propose a generic mapping method for landscape perception calculations. Figueroa-Alfaro et al. [30] used location photo data to evaluate Nebraska for landscape hotspot areas and aesthetic values. The focus of research on landscape perception has gradually shifted from studies that simply focus on the visual landscape itself to studies on the relationship between the visual landscape and other tourism variables. Wang et al. [31] revealed the relationship between visual landscape display and tourism experience in ancient villages by dividing the visual landscape, while Zheng et al. [32] studied the relationship between landscape perception and other variables in terms of tourist satisfaction and tourist perception.

Tourists will encounter a variety of emotional experiences during the tour, such as happiness, disappointment, excitement, anger, regret, etc. The factors that cause tourist disappointment mainly include poor service attitudes, bad weather, scenic congestion, unforeseen disasters, etc. Many scholars have studied tourist emotions at different types of tourist destinations from different perspectives. Enrique Bigné et al. [33] proposed a conceptual framework based on cognition, emotion, and satisfaction to analyze the perceived experiences of theme park visitors. Mehra et al. [34] used machine learning models to compute emotions from visitor UGC data. Liu et al. [35] separated and analyzed the domestic and foreign research on tourists’ emotional experiences from the perspectives of the influencing factors, dynamic changes, and the impact produced by tourists’ emotions. Xie et al. [36] combined the research of foreign tourism scholars and proposed a model of “one element, two poles and multiple factors” of tourists’ emotional experience from the aspect of the bipolarity of emotion. Liu et al. [37] summarized the spatial and temporal evolutionary characteristics of tourists’ emotions at special events.

## 3. Research Methods and Data Sources

### 3.1. Study Area Overview

**Figure 1:**
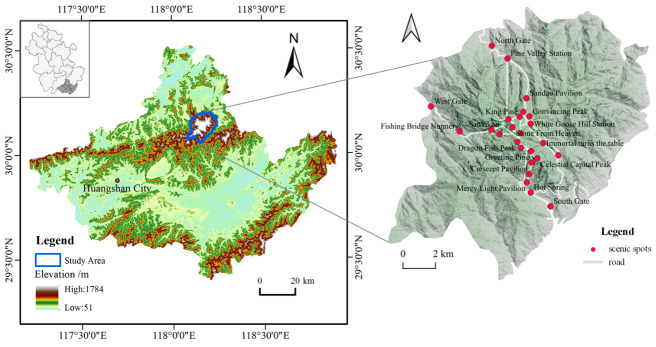
Schematic diagram of Huangshan Mountain.

Mountainous scenic spots are important factors for tourism, and their beautiful natural scenery and good ecological resources are the main aspects that attract tourists who wish to conduct tourism activities such as sightseeing, leisure and vacation, recreation, and health. In addition to natural tourism resources, most mountainous scenic spots also have profound cultural connotations and rich cultural ties, resulting in numerous cultural mountains that have existed throughout history. The Huangshan Mountain Scenic Area is located in the south of Anhui Province, with a scenic area of about 154 km^2^. It has been given a variety of accolades, such as “one of the top ten famous scenic spots in China”, “World Cultural and Natural Heritage”, “World Geological Park”, and “the first batch of 5A tourist attractions in China” [38] and has rich and colorful landscapes and good scenic services, which play a pivotal role in attracting tourists and generating income for the local area. The pine trees, the unpredictable sea of clouds, the rocky peaks, and the unique hot springs have become the core attractions of Huangshan for tourists [39]. As the most famous mountainous scenic spot in China, Huangshan attracts tourists from home and abroad, receiving an average of 3.38 million tourists annually, and this trend has been growing, with annual tourism revenue of CNY 2.9 billion [40]. Taking the Huangshan Mountain Scenic Area as a case study, we constructed a study on tourists’ landscape perceptions and emotional preferences regarding mountainous tourism scenic areas (Figure 1).

### 3.2. Data Source and Processing

The location photo data used in this study were crawled by using the Fiddler packet capture software and the “Houyi” collector crawler software on a “2BULU” outdoor travel open platform, with “Huangshan Scenic Area” and “Huangshan Mountain” as keywords. The platform web page json data packets were crawled by writing a Python program to parse the json packets, resulting in a total of 15,168 geotagged photo data containing URL links being obtained.

To ensure the accuracy of the data, this study preprocessed the photo data. Firstly, photos with an upload time that was earlier than its shooting time were deleted, and data redundancy was avoided by deleting photos of multiple locations taken by users at the same time, and secondly, only one photo was retained from a series of multiple photos taken by users at the same time; after pre-processing, 15,136 geotagged photos taken by tourists from 2014 to 2022 were ultimately retained as the base data for this study.

### 3.3. Research Methods

**Figure 2:**
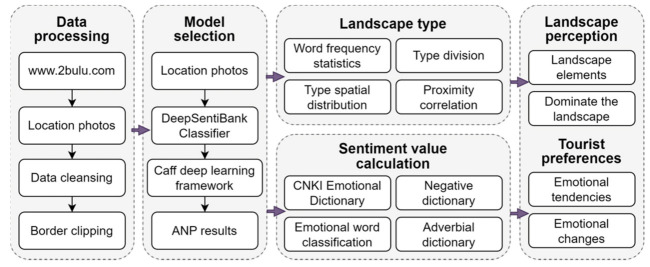
Research framework for visual perception and the emotional analysis of tourists (https://www.2bulu.com/ accessed on 6 December 2022).

We propose a research framework for mining the visual semantics of location photo data to analyze tourists’ landscape perception and sentiment preferences (Figure 2). The research framework utilized free and open location photo data for the visual semantic information extraction of the Huangshan Scenic Area and extracted ANP (adjective noun pairs) results of Huangshan tourists’ photos using the DeepSentiBank classification model combined with the Caffe deep learning framework [41,42,43]. The nouns in ANP are used to classify tourists’ landscape perceptions into nine types, and the adjectives in ANP, after processing, are combined with an emotion dictionary and a negation dictionary for the calculation of tourists’ emotional values. The integration of landscape perceptions and emotional values of tourists at Huangshan on temporal and spatial scales can better explain the visual landscape perceptions and the emotional preferences of tourists at Huangshan.

The research framework consists of four parts. The first part performs operations such as cleaning, processing, and boundary cropping on the collected location photo data. The second part uses the DeepSentiBank model to extract ANP results from the visual semantics of the location photos posted by tourists. The third part is as follows: ① Landscape type classifications of the extraction results of ANP of tourists’ photo data are performed, word frequency statistics on different landscape types are uncovered, the spatial distribution characteristics of each type are summarized, and the change characteristics of landscape types under different time and space scales are explored; ② natural language technology, a CNKI emotion dictionary, a negation dictionary, and a degree adverb dictionary are used to calculate the emotional value of photo data and the change characteristics of tourists’ emotions on different time and space scales are analyze. The fourth part: ① the landscape perception characteristics and the dominant landscape types on the attraction scale are analyzed; ② the emotional tendencies and emotional change trends of tourists at different time and space scales are analyzed.

This paper mainly adopts the method of the spatial gridding of data to represent data with an uneven geospatial distribution and carries out statistical analysis according to the size of the attributes and values of the data linked to the grid so as to clearly resolve the coupling characteristics of data and geospatial distribution in the study area. In this paper, a 150 m × 150 m grid was set up as the research unit, and the types of landscape perceptions and the magnitude of the emotional values of tourists’ photos in the grid at different spatial and temporal scales were statistically analyzed to explore the differences in the spatial and temporal distribution of tourists and the characteristics of their emotional preferences.

#### 3.3.1. DeepSentiBank

**Figure 3:**
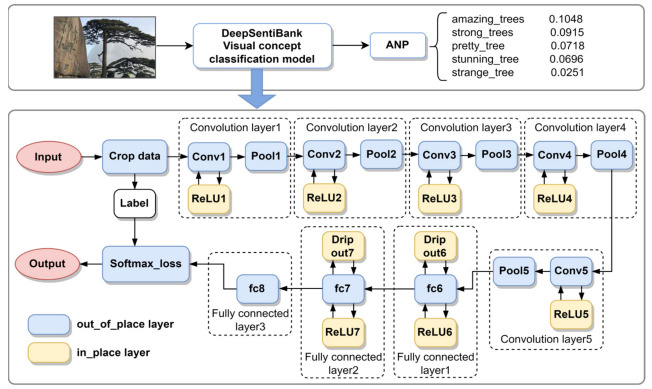
DeepSentiBank model with convolutional neural network structure.

In this paper, DeepSentiBank, a convolutional neural network-based visual emotion concept classification model, was used to mine the visual semantics in tourists’ photos (Figure 3). The model, proposed by Chen et al. at Columbia University [44], is based on the deep learning framework Caffe, which uses nearly one million Flickr images containing geotagged information to train a concept classifier that can be used to detect the visual content of images, and the recognition results of the photos are presented in the form of “adjective_noun”. The recognition results of photos are thus presented in the form of “adjective noun pairs” (ANPs), where nouns are visual concepts of images that can characterize the content of photos, and the model ranks 2089 ANPs according to the highest to lowest content confidence after recognizing an image.

The DeepSentiBank model is composed of a visual sentiment ontology, a large detector library, and a visual sentiment test benchmark and is a method based on psychological theory and web mining. A concept classifier trained on over 1 million geotagged photos is able to generate 2089 ANPs (adjective_noun pairs), consisting of 231 adjectives and 424 nouns, to transform picture information into text. This method can be used for picture sentiment prediction and is a systematic data-driven approach where the visual sentiment ontology consists of user-generated content based on the well-known psychological theory Plutchik’s Wheel of Emotions as a guiding principle and a mid-level visual representation that provides an automatic detector for discovered concepts and automatically infers the sentiment reflected in the image. In this paper, we used the DeepSentiBank model to analyze the content of tourist photos in the Huangshan Scenic Area, count the number of occurrences of ANP in each tourist photo, and retain the first five ANPs in each tourist photo to express the landscape that tourist paid attention to. The nouns in the first five ANPs of each photo were used to classify the photos into landscape types and word frequency statistics; the adjectives in the first five ANPs of each photo were used to calculate sentiment and analyze tourists’ emotional tendencies.

#### 3.3.2. Kernel Density Analysis

Kernel density analysis is an algorithm used to calculate the density of spatial distribution of point data [45], which does not need to assume the spatial distribution pattern of sample data in order to analyze and display the data of point distribution. Huangshan tourists’ photo data can be regarded as point data, so this paper uses the kernel density analysis method to analyze the photo data taken by Huangshan tourists and visualize and express the spatial distribution characteristics of tourists’ visual perception by setting the appropriate search radius and the size of output image elements.

$$f_{h}(x)=\frac{1}{nh}\sum_{i=1}^{n}k(\frac{x-x_{i}}{h})$$

Here, *f_h_(x)* is the kernel function, *x* − *x_i_* is the distance between the points to be estimated and the sample points. *h* is the radius, and *n* indicates the number of sample points.

## 4. Results and Analysis

### 4.1. Photo Visual Semantic Word Frequency Statistics

The DeepSentiBank model was used to parse the photos of tourists in the Huangshan Scenic Area, calculate the number of occurrences of each ANP, parse multiple results for one photo, and keep the top five ANP results as the recognition results of the photos in this study, which represent the types and frequencies of the landscapes that tourists pay attention to and help to analyze the landscape perception and emotional preference of tourists.

According to the results of the DeepSentiBank analysis and processing of tourist photos of Huangshan Mountain, the top five ANP result forms are output; the word frequency statistical analysis of the identified photos, the high-frequency words of ANP, and adjectives and nouns ranked in the top 30 most frequent words are listed; and the results of the word frequencies of ANP are analyzed, such as “dangerous roads”, “misty mountains”, “bright scenery”, “stunning mountains”, and “cloudy canyons” as the top ranking adjective_noun forms; the top ranking adjectives are “wet”, “ancient”, “cloudy”, “treacherous”, “dangerous”, and “natural”; and the top ranking nouns are “roads”, “scenery”, “mountains”, “forests”, “trees”, “canyons”, “bridges”, “palaces”, etc. It can be seen that the natural landscape of the Huangshan Scenic Area is the main concern of tourists, where strange pines, strange rocks, and dangerous peaks are the dominant landscape type.

Referring to the classification method of photos by Stepchenkova [8] and others, the photos of the Huangshan Scenic Area were classified into nine categories, namely mountain rocks, meteorology, hydrology, plants, animals, people, natural scenery, road facilities, and architecture, and the typical words associated with the nine types were summarized separately to analyze the types of attractions and visual perceptual interest points that tourists pay attention to during their visits (Table 1).

Of the visual semantic division of the recognition results of tourist photo points in the Huangshan Scenic Area, through the dimensional matching of all photo recognition results, word frequency statistics, and weight calculations of nine types of landscape images, the three types with the highest number of photos are mountain rocks (19.69%), road facilities (15.36%), and plants (13.23%); followed by architecture (12.22%), natural scenery (11.53%), people (10.61%), and meteorology (10.04%). The types with the smallest number of tourist photos are hydrology (4.73%) and animals (2.59%), both comprising less than 5%. The above data reflect that the main concerns of tourists’ photos are landscape types, such as mountain rocks, plants, etc., indicating the strong attractiveness of the Huangshan Scenic Area’s strange peaks, strange rocks, famous pines, etc.

### 4.2. Spatial Distribution of Photo Types

**Figure 4:**
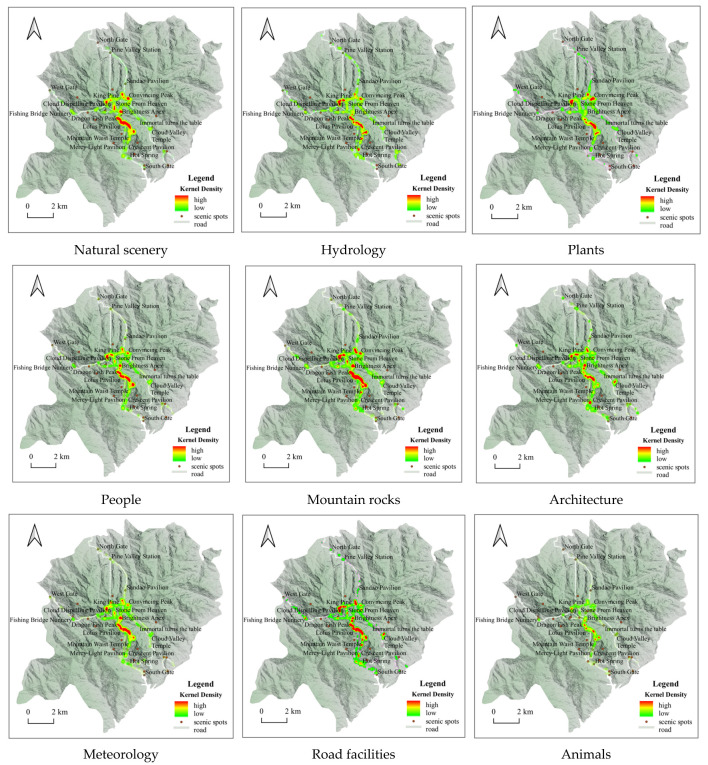
Kernel density analysis of landscape types of photographic interest to tourists.

For the nine types of mountain rocks, meteorology, hydrology, etc., a search radius of 100 m for kernel analysis was set. Hydrology and animals landscape perceptions generally showed the spatial characteristics of “scattered distribution” [46]; plants, architecture, and meteorology landscape perceptions generally showed the spatial characteristics of “extreme core significant”; mountain rocks, road facilities, natural scenery, and people landscape perceptions generally showed the spatial characteristics of “concentrated into a belt” (Figure 4).

### 4.3. Tourists’ Photo Landscape Perception

#### 4.3.1. Tourists’ Photo Landscape Perceptions at Different Time Scales

**Figure 5:**
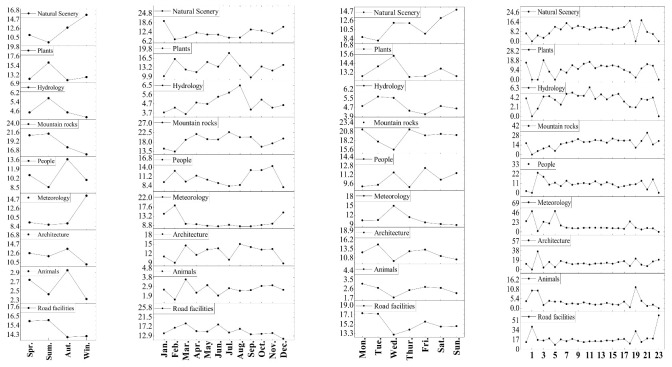
Tourists’ photos focus type on a scale of seasons, months, weeks, and hours.

The types of landscape perceptions of tourists visiting the Huangshan Scenic Area show significant imbalances in terms of the seasonal, monthly, weekly, and hourly time scales. Seasonal scale: Huangshan tourists’ landscape type perception shows different results during different seasons. The proportion of tourists’ landscape perceptions of mountain rocks and road facilities is higher in spring; the proportion of tourists’ landscape perceptions of mountain rocks and plants is higher in summer; the proportion of tourists’ landscape perceptions of mountain rocks and people is higher in autumn; the proportion of tourists’ landscape perceptions of mountain rocks, meteorology, natural scenery, and road facilities is higher in winter. As one of the most popular mountainous scenic spots, the landscape type that tourists pay the most attention to throughout the four seasons is mountain rocks. In summer, dense vegetation and hundreds of flowers bloom, which becomes a strong tourist attraction, and tourists have a strong perception of plants. The snowy scenery of Huangshan in winter is beautiful and invites tourists to stop and take photos. In addition to photographing mountain rocks, tourists also pay special attention to the meteorological landscape. Monthly scale: Mountain rocks are the main landscape type of concern for tourists throughout all months of the year; May–July is when tourists focus on plant types, August–November is when tourists focus on building types, and December–February is when tourists focus on meteorology, which is related to the snowy scenery of Huangshan. Weekly scale: The content of tourists’ photos was analyzed, and it was found that tourists’ attention to Huangshan Mountain on a weekly scale was focused mainly on mountain rocks, and the photos focusing on mountain rock types accounted for about 20% of the total, which greatly exceeded the proportion of any other type of photo. This may be related to the strange peaks and rocks scattered throughout the scenic area of Huangshan Mountain. Hourly scale: The hourly scale of tourists’ photo content was analyzed, and the results found that tourists were mainly concerned with the meteorological landscape type during the period of 03:00–06:00, and tourists mainly viewed landscapes related to sunrise during this time period; during the daytime period of 07:00–18:00, tourists’ behavior was mainly focused on the mountain rocks; at 19:00, tourists were focused on facilities, and tourists mainly returned to their hotels to rest or eat; at 23:00, photos are mainly focused on the facility type of landscape, as tourists are rest in their hotels (Figure 5).

#### 4.3.2. Tourist Photo Landscape Perceptions at Different Spatial Scales

**Figure 6:**
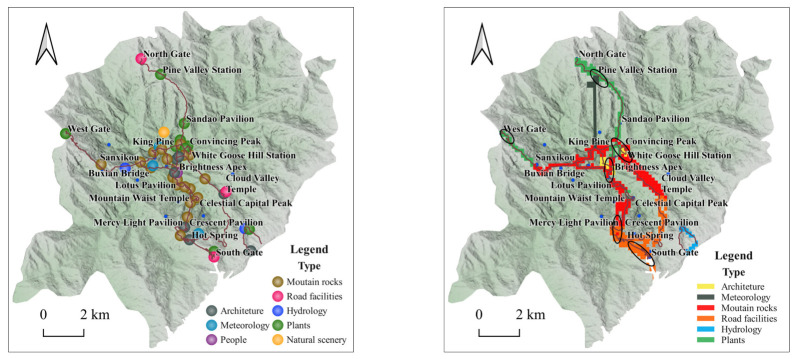
Tourists’ photos focus in terms of scenic spots and areas.

The landscape perception of tourists’ photos in the Huangshan Scenic Area demonstrates obvious aggregation characteristics on the spatial scale of attraction and scenery. Scales of attractions: Tyson polygons are established with the attractions as the center, and the attributes of the attractions are assigned to the Tyson polygons through spatial links, and then the Tyson polygons attributes are assigned to all photo point data using spatial links; the type with the most number of photos in the scope of each attraction is counted as the dominant landscape type of the attraction. From the data analysis, we can see that the dominant landscape type that tourists pay attention to regarding different attractions are mountains and rocks, accounting for 43% of the total number of photo types. Scenery scale: According to the statistics of tourists’ photo types in the Huangshan Scenic Area, the dominant landscape types that tourists pay attention to in the different parts of the scenic area are mostly mountain rocks, which is in line with the characteristics of the Huangshan Scenic Area, and the dominant landscapes that tourists pay attention to are basically consistent with the recommendations of the Huangshan Scenic Area Management Committee. The hydrological type mainly appears in the area of Nine Dragons Waterfall, which is related to the fact that the Nine Dragons Waterfall is the most famous water body in the Huangshan Mountain Scenic Area; the road facility type mainly appears in the South Gate—Hot Spring section, which is the location where tourists enter and exit the scenic area, and the Hot Spring—Cloud Valley Temple and Hot Spring—Mercy Light Temple, which is an important area where tourists can choose to change to the ropeway; the plant type mainly appears in Mountain Waist Temple—Greeting Pine, Celestial Capital Peak—Greeting Pine, North Gate—Refreshing Terrace, and West Gate—Fishing Bridge Nunnery, as the pine trees in these sections are of various types and strange shapes, so they are the places where tourists take the most pictures of pine trees (Figure 6).

### 4.4. Tourist Photo Emotional Preferences

#### 4.4.1. Emotional Preferences of Tourists’ Photos at Different Time Scales

**Figure 7:**
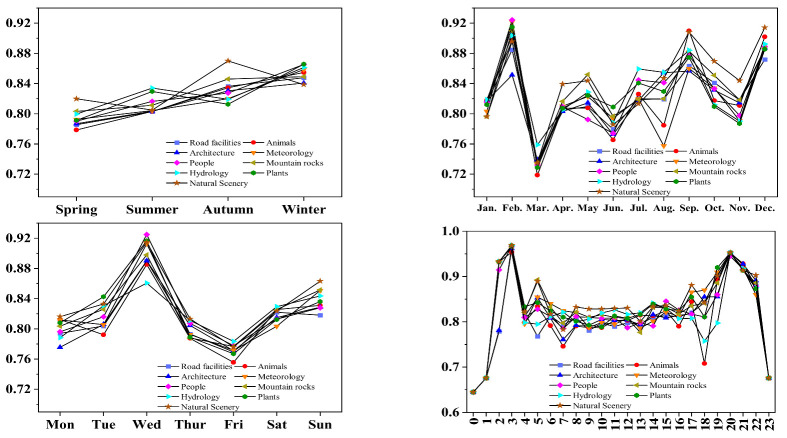
Emotional value of tourists’ photos on a scale of seasons, months, weeks, and hours.

Photographs of tourists’ emotions in the Huangshan scenic area show a significant fluctuation in terms of seasonal, monthly, weekly, and hourly time scales. Seasonal scale: The distribution of seasonal emotional values of tourists’ photos is the “slowly sloping straight line” type, and the emotional values of tourists in nine types of landscapes show different changes with the seasons. The order of emotional value size is the following: spring emotional value < summer emotional value < autumn emotional value < winter emotional value. Spring tourists have the highest emotional value regarding mountain rocks and natural scenery and the lowest emotional value for animals and road facilities; summer tourists have the highest emotional value in terms of hydrology and plant-type landscapes and the lowest emotional value for road facilities, animals, and buildings; autumn tourists have the highest emotional value for mountain rocks and natural scenery and the lowest emotional value for hydrology and plant landscapes; winter tourists have a higher overall emotional value for photos, including plants and people. The highest sentiment value is for winter photos. Monthly scale: The distribution of the emotional value of Huangshan tourists in the month is the “W” type; the average emotional values in February, September, and December are high, and the average emotional values in March, June, and August are low. June belongs to the rainy season in Huangshan, and thus frequent rainfall affects tourists’ visiting experience; August coincides with holidays, which is related to crowded visits. The lowest sentiment value of landscape is the animal type, and the lowest sentiment value of landscape in August is the meteorology type. Weekly scale: Huangshan tourists’ weekly emotional value distribution is the “N” type. Wednesday’s emotional value is the highest, Friday’s emotional value is the lowest, and the nine landscape types of tourists’ emotional value change trends are basically the same. Hourly scale: Huangshan tourists’ hourly emotional value distribution is the “M” type, 03:00 and 20:00 are the highest emotional value times of the day, 00:00 and 23:00 are the lowest emotional value times of the day, and the trends of tourists’ emotional value in terms of the nine types of landscapes are basically the same (Figure 7).

#### 4.4.2. Emotional Preferences of Tourists’ Photos on Different Spatial Scales

**Figure 8:**
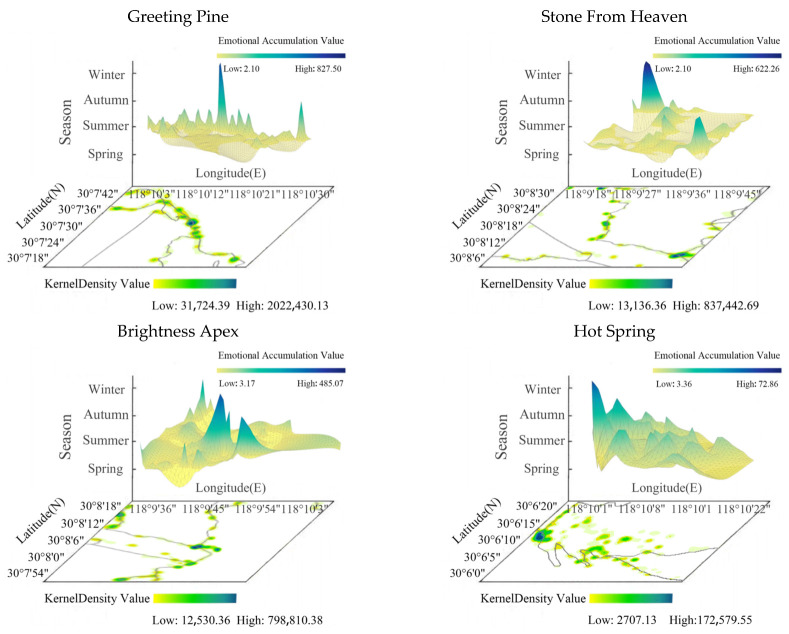
Popular tourist emotional value zones at Greeting Pine, Stone From Heaven, Brightness Apex, and Hot Spring.

The high value of tourists’ photo emotions in the Huangshan scenic area at the spatial scale shows obvious aggregation characteristics. The high values of photo emotions are mainly distributed in the vicinity of Convincing Peak—North Sea, Celestial Capital Peak—Lotus Peak, and Hot Spring. These places are the concentrated distribution areas of the iconic landscape of the Huangshan Mountain Scenic Area with strange pines (Greeting Pine), strange rocks (Stone From Heaven), sunrise (Brightness Apex), and hot springs (Huangshan Hot Spring), which provide a strong attraction and experience for tourists (Figure 8).

In this section on high emotional values on a spatial scale in photographs, it is important to identify not only the distribution of popular zones of emotional value at the iconic attractions of Huangshan Mountain (Greeting Pine, Stone From Heaven, Brightness Apex, and Hot Spring) but also the evolution of the expansion and contraction of tourists’ emotional values over time (seasons). In this study, the distribution of the seasonal scale tourist emotional zones at iconic attractions was identified by dividing the attractions into grid cells, using the grid cells to represent the cumulative emotional values of the photographs, and then using ArcGIS to calculate the kernel density values of the associated areas and characterize the diffusion and contraction of the tourists’ emotional zones though natural breakpoints in order to generate a heat zone map of the iconic attractions.

**Figure 9:**
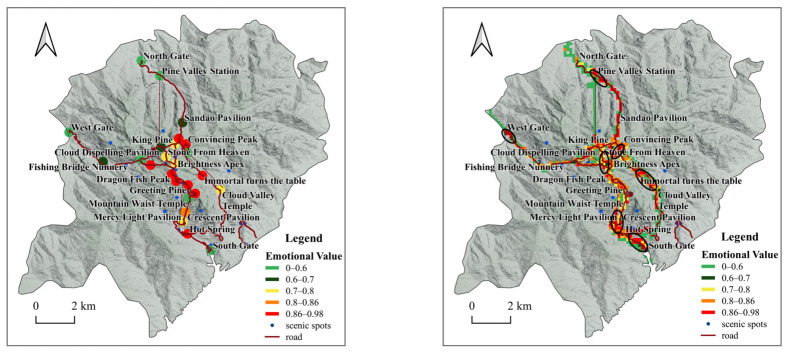
Emotional value of tourists’ photos on a scale of scenic spots and areas.

Scales of attractions: Taking attractions as spatial scales, the number of photos taken by tourists at each attraction of Huangshan Mountain was counted with the amount of emotional value. The number of photos taken by tourists at different attractions varies widely, with the most number of photos taken at the Greeting Pine, Jade Screen Station, Lotus Pavilion, Tihai Pavilion, and Celestial Capital Peak, while fewer photos are taken at the entrance of the North Gate and West Gate, which is related to the low number of tourists entering and leaving the scenic spot from this location. The sentiment values of the attractions differ widely, with tourists having higher sentiment values at White Goose Lodge and lower sentiment values at Nine Dragons Pavilion. Emotional value calculation with attractions as a scale demonstrates that the emotional value of attractions at the entrances of North Gate, West Gate, and South Gate is low, and the emotional value at the location of the transfer area is even lower due to the long waiting times for tourists at the ropeway floor track, and the crowds and queues resulting in lower emotional values; the higher emotional value at popular attractions, such as Mountain Waist Temple, Greeting Pine, and Celestial Capital Peak, is related to the positive experiences of the tourists. Scenic section scale: The number of photos taken by tourists in each scenic section around Huangshan Mountain and the magnitude of emotional value is counted. It was found that the highest tourist sentiment value is distributed in Dragon Fish Peak—Heaven Sea and Refreshing Terrace—North Gate, and the lowest tourist emotional value is distributed in Nine Dragons Waterfall—Nine Dragons Pavilion, Cloud Dispelling Pavilion—King Pine, and Cloud Valley Temple—White Goose Hill. The highest number of photos taken by tourists are distributed in White Goose Hill—North Sea, Lotus Pavilion—One-Line-Sky, West Sea—Heaven Sea, Celestial Capital Peak—Greeting Pine, and the lowest number of photos taken by tourists are distributed in Dragon Fish Peak—Heaven Sea, North Sea—Refreshing Terrace, Refreshing Terrace—North Gate, and Jade Screen Ropeway (Figure 9).

The high emotional value of the Huangshan Mountain tourist photo scenery scale is mainly concentrated in the scenic area entrances and exits, ropeways, the transfer area for the ground rail, and high-quality, well-known attractions in several areas. Tourists enter the scenic area when energetic emotional value is high; the ropeway and other public facilities can save energy, and visitors can focus on viewing the landscape of Huangshan; well-known attractions can provide tourists with a better tourist experience, so the emotional value is high.

## 5. Discussion

Location photo data reflect tourists’ instinctive concerns and feelings about the landscape, the visual semantic content of the photos can be used to explore the types of landscape and emotional preferences of tourists’ concerns, and the location and time of the photos can be used to explore the spatial and temporal distribution characteristics of the types of tourists’ landscape perceptions and the resources they rely on [47]. Tourists’ location photos have the advantages of large data volumes and easy access. Therefore, using photo data to carry out research on tourists’ landscape perceptions and affective preferences will become an important method of achieving these data.

Computer image recognition technology and emotion prediction methods are able to effectively solve the problems of accuracy and large data volumes of image recognition and can effectively advance research on tourists’ landscape perceptions and emotional preferences [48]. Existing studies are mostly limited by the data sample size and visual semantic processing techniques and are not able to comprehensively and quantitatively uncover the characteristics of tourists’ perceptions of scenic landscapes. This study uses DeepSentiBank to solve these problems using image recognition and sentiment analysis with large data volumes [42,49], which is an effective method that is able to identify the emotions in photos [44]. Mountain scenic areas have unique natural conditions, and it is easy to undertake various activities, such as mountaineering, adventure, science, research, etc., while giving full play to its natural resources, thus improving the number of landscape types that tourists can enjoy, resulting in higher visitor satisfaction [50], while also tapping into an area’s cultural resources. Taking the mountainous scenic area of Huangshan as an example, the following conclusions were drawn by mining the semantic information of scenic tourists’ photo data and exploring the differences in the types of landscape perceptions and emotional preferences of tourists at different time scales and spatial scales.

(1) Through the visual semantic analysis of tourists’ photos in Huangshan Mountain, we were able to obtain nine types of themes, namely mountain rocks, road facilities, plants, architecture, natural scenery, people, meteorology, hydrology, and animals, in order according to the number of photos, accounting for 19.69%, 15.36%, 13.23%, 12.22%, 11.53%, 10.61%, 10.04%, 4.73%, and 2.59%, respectively. According to the emotional worth score, tourists’ photo emotions were classified into five ranges (0, 0.6), (0.6, 0.7), (0.7, 0.8), (0.8, 0.86), and (0.86, 0.98).
(2) The spatial differentiation of tourists’ visual landscape perceptions: ① From an overall perspective, the perception of hydrology and animals landscape images generally has the spatial characteristics of “scattered distribution”, and plants, architecture, and meteorology landscape perception images in general present “significant nucleus” spatial characteristics. The perceived images of mountain rocks, road facilities, natural scenery, and people display spatial distribution characteristics of “concentrated into a belt”. ② From the perspective of scenic spots, Stone From Heaven, Brightness Apex, Celestial Capital Peak, etc., belong to the photographic theme of rock images; Cloud Valley Temple Station, South Gate, North Gate, etc., belong to the theme of road and facilities images; King Pine, Convincing Peak, Black Tiger Pine, etc., belong to the theme of plant images; Mercy Light Temple, Cloud Valley Temple, Sea Heart Pavilion, etc., belong to the theme of architectural images. ③ The emotional value of North Gate, South Gate, Pine Valley Station, West Gate, and Mountain Waist Temple is less than 0.65, while the emotional value of Celestial Capital Peak, Dragon Fish Peak, Convincing Peak, and Greeting Pine is higher, namely between 0.65 and 0.85. A characteristic analysis of cumulative emotional value was conducted for the representative attractions recommended by the Huangshan Tourism Marketing Organization, namely Stone From Heaven, Brightness Apex, Greeting Pine, and Hot Spring, and it was found that the cumulative emotional value of Hot Spring is much lower than the other three attractions, which is inconsistent with the recommended results of the Huangshan Tourism Marketing Organization. Hot Spring is the first stop when entering the scenic area from the gate of Huangshan Mountain, and few tourists soak in Hot Spring first before climbing the mountain. In addition, Hot Spring needs to pay higher fees, which may lead to a low emotional accumulation value for tourists. Therefore, it is necessary to add cultural design and implement landscape improvements to Huangshan Hot Spring, improve the marketing strategy and service measures of Hot Spring, and provide reasonable promotion and guidance to tourists so as to enhance the tourism quality of Hot Spring.
(3) In terms of the temporal divergence of landscape types of concern in tourists’ photos: ① From the seasonal scale, mountain rocks are the landscape type of concern for tourists throughout all seasons. In addition, in summer, visitors also pay attention to plant landscape types, and in winter, they pay attention to meteorology, natural scenery, and roads and facilities. The change in tourists’ emotional value on the seasonal scale is a “slowly sloping straight line” type, and tourists’ emotional value is highest in winter and lowest in spring. ② On the monthly scale, throughout all months, tourists focus on the mountain rock landscape type; in December–February, they focus on the meteorology landscape type; and in May–July, they focus on the plant landscape type. The change in tourists’ emotional value on the monthly scale is in the shape of a “W”, with the highest emotional value in February and the lowest emotional value in March. ③ On the weekly scale, the mountain rock landscape is the type that attracts attention seven days a week. The change in the emotional value of tourists on the weekly scale is the “N” type. ④ On an hourly scale, the meteorology landscape is the type that tourists mainly pay attention to from 03:00 to 06:00. The change of tourists’ emotional value varies on the hourly scale in the shape of “M”, with the highest emotional value at 03:00 and the lowest at 00:00.

The “2BULU” platform data used in this paper was not able to accurately obtain the attribute information of tourists, such as gender, which limits the mining of different types of tourists’ landscape perceptions and affective preferences. In addition, tourists’ landscape perceptions are related to other factors besides visual carriers, so we should continue to expand the variety of data sources in the future to build a complete model of tourists’ landscape perceptions and affective preferences and supplement it with a multi-case comparison [51]. Location photos are rich in semantic information but have limitations in reflecting complex emotional preferences, especially when it comes to tourists’ complex emotions. Emotions are generated by tourists as they enjoy landscapes and are present in nature. Photographs are a small way of recording tourists’ emotions and behaviors; however, it is difficult to reflect tourists’ landscape perceptions and complex emotional preferences comprehensively and completely, and in addition, it is difficult to reflect the scenes of tourists’ emotional interactions with local residents and scenic area service personnel during the tour.

## 6. Conclusions

In this paper, using deep learning convolutional neural network image recognition technology and taking the Huangshan Scenic Area as an example, we used the DeepSentiBank image recognition model with a quantitative calculation of tourists’ emotional values to establish a photo visual semantic mining method to quantify landscape perceptions and tourists’ emotional preferences and attempt to analyze the influencing factors of variance from multiple perspectives. Nine types of tourists’ preferred landscape types are summarized herein, and it was found that tourists pay the most attention to mountain and rock landscapes and the least attention to animal landscapes; the distribution of tourists’ high emotional value on different spatial scales differs significantly. The high emotional value regarding attractions is mainly distributed near the iconic attractions, and the high emotional value on the scenery scale is mainly distributed around the entrances and exits of the Huangshan Scenic Area. Parsing the visual semantic information conveyed by tourists’ photos can provide a reference for tourism destination marketing organizations to design marketing strategies for different types of tourists. The visual semantic analysis of photos can reveal the landscape perceptions and emotional preference characteristics of tourists on different spatial and temporal scales, which helps the daily management and emergency warnings of scenic spots and is conducive to the formulation of tourist tour routes during peak hours and holidays, and thus to the creation of sustainable tourism products and appropriate routes based on the types of landscapes of interest to tourists at different times in order to ensure tourism safety and promote the high-quality development of mountainous scenic spots.

## Figures and Tables

**Table 1:** Huangshan tourists’ photos focus type.

Type	Noun
Mountain rocks	bridge, fence, garden, hill, hills, mountain, mountains, tower, valley
Meteorology	sun, air, autumn, beach, clouds, darkness, dawn, fog, ice, island, mist, moon, morning, night, places, rain, rainbow, sea, sky, snow, spring, star, storm, summer, sunlight, sunrise, sunset, waves, winter
Hydrology	spa, bay, creek, lake, pond, pool, river, water, waterfall, stream
Plants	blossom, flora, flower, flowers, forest, grass, leaves, mushrooms, plant, rose, shadow, shadows, tree, trees, wood, woods
Animals	animal, animals, bat, bats, bird, birds, bull, butterfly, cat, cats, chicken, cockroach, creatures, deer, dog, fish, fishing, hawk, horse, insect, kitty, monkey, pet, pets, pig, pony, puppy, rabbit, snake, spider, wings, wolf
People	actor, adult, artist, baby, band, boy, chest, child, childhood, children, dad, driver, eyes, face, family, fan, friends, girls, glasses, guard, hands, hat, head, heart, kids, lady, legs, lips, men, mothers, mouth, parents, police, soldier, student, team, tears, teen, volunteers, worker
Natural Scenery	earth, island, landscape, moon, nature, places, reserve, scene, scenery, sky, sunlight, view, views, wonder
Road Facilities	road, street, streets, hospitals, hotel, bed, phone, piano, places, theatre, tour, toy, train, vacation, vehicle, wood
Architecture	architecture, building, painting, paintings, statue, architecture, backyard, building, castle, chair, church, construction, design, farm, fence, fortress, hall, heritage, history, landmark, sign, space, toilet, tower, wall, window, sculpture, statue

## Data Availability

The data used to support the findings of this study are included within the article.
